# Functional and structural connectome properties in the 5XFAD transgenic mouse model of Alzheimer’s disease

**DOI:** 10.1162/netn_a_00048

**Published:** 2018-06-01

**Authors:** Shelli R. Kesler, Paul Acton, Vikram Rao, William J. Ray

**Affiliations:** Department of Neuro-oncology, University of Texas MD Anderson Cancer Center, Houston, TX, USA; Neurodegeneration Consortium, Institute for Applied Cancer Science, University of Texas MD Anderson Cancer Center, Houston, TX, USA; Department of Neuro-oncology, University of Texas MD Anderson Cancer Center, Houston, TX, USA; Neurodegeneration Consortium, Institute for Applied Cancer Science, University of Texas MD Anderson Cancer Center, Houston, TX, USA

**Keywords:** Alzheimer’s disease, Connectome, Neuroimaging, fMRI, Diffusion tensor imaging

## Abstract

Neurodegeneration in Alzheimer’s disease (AD) is associated with amyloid-beta peptide accumulation into insoluble amyloid plaques. The five-familial AD (5XFAD) transgenic mouse model exhibits accelerated amyloid-beta deposition, neuronal dysfunction, and cognitive impairment. We aimed to determine whether connectome properties of these mice parallel those observed in patients with AD. We obtained diffusion tensor imaging and resting-state functional magnetic resonance imaging data for four transgenic and four nontransgenic male mice. We constructed both structural and functional connectomes and measured their topological properties by applying graph theoretical analysis. We compared connectome properties between groups using both binarized and weighted networks. Transgenic mice showed higher characteristic path length in weighted structural connectomes and functional connectomes at minimum density. Normalized clustering and modularity were lower in transgenic mice across the upper densities of the structural connectome. Transgenic mice also showed lower small-worldness index in higher structural connectome densities and in weighted structural networks. Hyper-correlation of structural and functional connectivity was observed in transgenic mice compared with nontransgenic controls. These preliminary findings suggest that 5XFAD mouse connectomes may provide useful models for investigating the molecular mechanisms of AD pathogenesis and testing the effectiveness of potential treatments.

## Author Summary

Many connectome properties have been shown to be preserved across species, providing potentially novel insights regarding the mechanisms of various disease processes. In this study, we measured functional and structural connectomes in a transgenic mouse model of Alzheimer’s disease using resting-state functional MRI and diffusion tensor imaging. We showed that connectome organization was significantly altered in transgenic mice compared with nontransgenic controls in ways that parallel what has been observed in human patients. These findings suggest that transgenic mouse connectomes may be useful for studying the etiology and treatment of Alzheimer’s disease.

## INTRODUCTION

Alzheimer’s disease (AD) is the most common form of age-related neurodegeneration and dementia (Risacher & Saykin, 2013). AD pathology initiates many years before diagnosis and develops slowly in some individuals and more rapidly in others. Patients with incipient AD initially are cognitively normal, but inevitably progress to severe dementia and death. Over 46 million people have Alzheimer’s dementia globally, and the prevalence is expected to double every 20 years (Prince et al., 2015). There currently are no effective treatments for reversing AD. Risk factors include age, first-degree family history, and the apolipoprotein E (APOE) e4 genotype (Green et al., 2002; Hebert et al., 2010; Saunders et al., 1993; ten Kate et al., 2016; Wolters et al., 2017). However, the only causative factors identified to date are mutations in amyloid precursor protein (APP), presenilin 1 (PSEN1), or presenilin 2 (PSEN2) genes. These mutations are rare but tend to be associated with aggressive, early onset disease and therefore have provided unique information regarding the pathophysiology of AD (Bateman et al., 2011).

AD is associated with significant amyloid-beta peptide accumulation, which is produced from APP by PSEN1 and PSEN2, leading to the hypothesis that it is a primary mechanism of neurodegeneration (Hardy & Selkoe, 2002; Lloret, Fuchsberger, Giraldo, & Vina, 2015). Neuroimaging studies of patients with AD demonstrate significant abnormalities in brain structure and function. These abnormalities are abundant in frontal and temporal regions, including the hippocampus and prefrontal cortex, but tend to reflect widespread disruption of large-scale, distributed networks.

In vivo functional neuroimaging of transgenic mice may yield important insights regarding the mechanisms of AD and provide preclinical models for testing the effectiveness of candidate drugs on preventing or reversing AD-related neuropathology. Previous studies of APP/PS1 and ArcBeta transgenic mice have demonstrated deficits in the functional connectivity of multiple brain regions that is associated with amyloid deposition (Bero et al., 2012; Grandjean et al., 2014; Shah et al., 2013). The five-familial AD (5XFAD) transgenic mouse model expresses three APP and two PSEN1 mutations on a (C57BL/6 x SJL)F1 background. These mice demonstrate accelerated amyloid deposition and have an early onset, aggressive disease presentation. They are particularly useful for investigating the effects of amyloid-beta deposition on neuronal loss (Eimer & Vassar, 2013).

Few if any studies have examined connectome organization in transgenic AD mice or any disease group. Connectomics models brain networks as graphs with nodes (regions) and edges (connections; Bassett & Bullmore, 2006). These mathematical models of brain networks provide measurements of information-processing efficiency and resilience to pathology, among other topological properties that are highly relevant to AD (Contreras, Goni, Risacher, Sporns, & Saykin, 2015; Dai & He, 2014; Tijms, Wink et al., 2013). Connectome properties have been shown to be preserved across species and therefore provide a unique translational bridge between preclinical and clinical studies (Gorges et al., 2017; Oh et al., 2014; Stafford et al., 2014; van den Heuvel, Bullmore, & Sporns, 2016).

Connectome studies of patients with AD have most consistently demonstrated alterations in measures of network integration and efficiency (Daianu et al., 2013; Fischer, Wolf, Scheurich, Fellgiebel, & Alzheimer’s Disease Neuroimaging Initiative, 2015; Kim et al., 2016; Lo et al., 2010; Pereira et al., 2016; Reijmer et al., 2013; Stam, Jones, Nolte, Breakspear, & Scheltens, 2007; Wang et al., 2012; Zhao et al., 2012). Additionally, studies suggest that AD pathogenesis targets high-traffic hub regions in the brain, spreading from epicenters to secondary networks as the disease progresses (Buckner et al., 2009; Dai et al., 2015; Mallio et al., 2015; Stam et al., 2009; Zhou, Gennatas, Kramer, Miller, & Seeley, 2012; Zhou & Seeley, 2014). We compared functional and structural connectomes of 5XFAD transgenic mice with those of nontransgenic controls. The aim of this pilot study was to determine whether 5XFAD mice show alterations in brain networks that parallel those observed in patients with AD, including elevated characteristic path length, reduced network efficiency, and decreased hub presence.

## METHODS

### Subjects

5XFAD mice were purchased from the Jackson Laboratory and maintained on the B6SJLF1/J background. Mice were maintained on a 12-hour light/dark cycle at room temperature of 75°F with unrestricted access to food and water. In total, eight male mice, 23 weeks of age, were used in this experiment. Four mice were 5XFAD transgenic and four were nontransgenic littermate controls. 5XFAD mice at 23 weeks of age have been shown to have cognitive deficits that are prior to significant neuronal or synaptic loss (Eimer & Vassar, 2013; Oakley et al., 2006). Our protocols were approved by the University of Texas MD Anderson Institutional Animal Care and Use Committee.

### Neuroimaging

We obtained in vivo resting-state functional magnetic resonance imaging (rsfMRI) data from mice using a 7 Tesla Bruker BioSpec (Bruker BioSpin, Billerica, MA) scanner while mice were anesthetized with isoflurane. Isoflurane was administered at 1% (mixed with O_2_) to keep the respiration rate between 80 and 120 beats per minute (Stafford et al., 2014). Mice were secured into the head coil with a bite bar and the head was taped down to minimize motion. We first acquired a single-shot gradient, axial echo planar imaging (EPI) functional sequence (slice thickness = 0.5 mm, gap = 0.0 mm, repetition time [TR] = 2,000 ms, echo time [TE] = 12 ms, matrix = 80 × 64 × 32, field of view [FOV] = 20 × 16 mm, flip angle = 75°, number of volumes = 450, averages = 1, scan time = 15 min) followed by a T2-weighted, turbo spin echo, rapid acquisition with refocused echoes (Turbo RARE) sequence (slice thickness: 0.5 mm, gap = 0.0 mm, TR = 4,000 ms, TE = 40.00 ms, matrix = 256 × 180, FOV = 26.600 × 18.000 mm, flip angle = 90°, number of images = 32, scan time = 4 min and 24 s).

Six days following rsfMRI mice were euthanized using carbon dioxide and transcardially perfused with 20 ml of 10 U/ml heparin (Sagent Pharmaceuticals, Schaumburg, IL) in PBS pH 7.4 (Invitrogen, Carlsbad, CA) at room temperature followed by 20 ml 4% paraformaldehyde in PBS pH 7.4 (Electron Microcopy Sciences, Hatfield, PA) at room temperature. Following perfusion, the heads were removed and the skin, muscle, eyes, ears, nose tip, and lower jaw were removed to expose the skull. The skulls were then immersed in 20 ml of 4% paraformaldehyde in PBS pH 7.4 overnight at 4°C with continuous mixing. The skulls were then transferred to 50 ml of 0.01% sodium azide (Teknova, Hollister, CA) in PBS pH 7.4 at 4°C for seven days with continuous mixing. At the end of the seven days the skulls were transferred to 50 ml of 5 mM Magnevist (Gadopentetate Dimeglumine; Bayer Healthcare Pharmaceuticals, Indianola, PA), 0.01% sodium azide in PBS pH 7.4 at 4°C for 24 days with continuous mixing. Following the Magnevist treatment the skulls were transferred to 50 ml of 0.01% sodium azide in PBS pH 7.4 at 4°C and maintained in this solution with continuous mixing until the day of imaging, when the skulls were transferred to Fomblin Y (Sigma-Aldrich, Saint Louis, MO). We then acquired ex vivo diffusion tensor imaging (DTI) data using a 9.4 Tesla Bruker Avance BioSpec scanner (fMRI was not available on this scanner at the time of this study). The following parameters were used: spin echo, b-value = 0 and 1,000 s/mm^2^, 20 diffusion directions with one non–diffusion weighted image, TR = 500 ms, TE = 14.8 ms, FOV = 17 × 12.5 × 15 mm, matrix = 180 × 133 × 160, NEX = 1, *δ* = 3 ms, Δ = 7 ms, scan time = ∼35 hr.

A brain mask was manually delineated in 3D for the T2 and rsfMRI volumes in FMRIB Software Library (FSL) View v3.2.0 to remove the skull. RsfMRI data were preprocessed in Statistical Parametric Mapping v8 including realignment and warping of the EPI volume via the co-registered T2-weighted volume to a male C57BL/6 mouse brain template (Ma et al., 2005). CONN Toolbox v13 software was then used to filter data to the <0.1 Hz range of spontaneous activity (Raichle, 2011; Whitfield-Gabrieli & Ford, 2012). CONN implements the CompCor method to remove motion and physiologic/nonneuronal artifacts. This method involves extracting signal from white matter and cerebrospinal fluid regions using principal component analysis and then regressing these signals out of the total fMRI signal (Behzadi, Restom, Liau, & Liu, 2007). Functional time series were extracted from each of 32 bilateral cortical and subcortical gray matter regions of interest to cover the entire brain (Supplementary Figure 1; Kesler, Acton, Rao, & Ray, 2018), cross-correlated and normalized using Fisher r-to-z transformation.

DTI preprocessing was performed in FSL v5.0 (Smith et al., 2004) including eddy current correction and tensor reconstruction. Deterministic tractography was performed in TrackVis v0.6.1 (Wang, Benner, Sorensen, & Wedden, 2007) using an FA threshold of 0.1 and a curvature threshold of 40°, based on the study by Chen et al. (2015). The 32 regions of interest described above were warped into DTI native space via inverse transformation of the b0 volume to the mouse brain template. We determined the number of DTI streamlines connecting each pair of regions, and regions were considered connected if one streamline endpoint terminated within one region and the other endpoint terminated within the other region. A threshold of three streamlines was applied to minimize false-positive streamlines, and each valid edge was weighted by the average streamline fractional anisotropy (Kesler, Watson, & Blayney, 2015).

Functional and structural connectomes were constructed for each participant with *N* = 32 nodes, network degree of *E* = number of edges, and a network density of *D* = *E*/[(*N* × (*N* − 1))/2] representing the fraction of present connections to all possible connections. Negative functional edges were zeroed given evidence that properties of negative correlation networks are different than those of positive correlation networks (Hosseini & Kesler, 2013; Schwarz & McGonigle, 2011). Structural connectomes were scaled to the range of 0 to 1 (Wang, Ghumare, Vandenberghe, & Dupont, 2017).

### Statistical Analysis

Connectome properties were calculated using graph theoretical analysis. Specifically, we measured characteristic path length and global/local efficiencies to test our hypothesis that these properties would be altered in transgenic mice consistent with studies of patients with AD. We additionally measured normalized clustering coefficient, small-worldness index, and modularity as these have also been reported in human studies of AD (Dai & He, 2014; Tijms, Wink et al., 2013). Connectome properties were defined as previously described (Bassett & Bullmore, 2006; Rubinov & Sporns, 2010; Sporns & Betzel, 2016). Briefly, characteristic path length is the average shortest path length between all pairs of nodes normalized by the characteristic path length of random networks. Normalized clustering coefficient is the proportion of actual connections to possible connections between a node’s neighbors normalized by the clustering coefficient of random networks. Small-worldness index is defined as normalized clustering coefficient/characteristic path length. Path length and clustering coefficient were normalized using 20 benchmark random networks (Zalesky, Fornito, & Bullmore, 2012). Global efficiency is the inverse average shortest path length of the network, while local efficiency is the inverse of the average shortest path connecting all neighbors of a node, or in other words, the average efficiency of the local subgraphs. Modularity analysis involves decomposing the network into nonoverlapping groups of regions (modules) that have maximal within-group connections and minimal between-group connections. Connectome measurement was conducted using Brain Connectivity Toolbox (Rubinov & Sporns, 2010).

Thresholding connectomes is necessary for removing false-positive edges and facilitating between-group comparisons but can remove potentially valid information regarding differences in network topology (Fornito, Zalesky, & Breakspear, 2013; van Wijk, Stam, & Daffertshofer, 2010). Further, there tends to be a large difference in network densities between rsfMRI- and DTI-derived connectomes. Therefore, we compared connectome properties across multiple densities using the area under the curve (AUC; Bassett, Meyer-Lindenberg, Achard, Duke, & Bullmore, 2006; Bassett, Nelson, Mueller, Camchong, & Lim, 2012). Specifically, we measured connectome properties at each density from minimum connection density to the last density associated with a small-world organization (Basset et al., 2008; Humphries & Gurney, 2008) up to a maximum density of 0.5 (Kaiser & Hilgetag, 2006). We then measured the AUC across this entire range as well as in a windowed manner where target windows were determined from visual inspection of the small-worldness index curves. This approach was based loosely on the clustering method introduced by Drakesmith et al. (2015). AUCs were compared between groups using nonparametric permutation testing (Basset et al., 2008) using 2,000 iterations and two-tailed *p* values.

We also evaluated weighted networks without any thresholding. Connectome properties from weighted networks were compared between groups using the general linear model with network density as a covariate (Brown et al., 2011). The weighted network data are provided in the Supplementary Information (Kesler et al., 2018).

To examine hub profiles, we determined whether the cumulative degree distribution of the networks followed an exponentially truncated power-law indicating the presence of hub regions (Achard, Salvador, Whitcher, Suckling, & Bullmore, 2006). This analysis was performed with weighted networks and at minimum density. Power-law fitting and comparison was conducted in the R statistical package v3.3.2 (R Foundation) using the “poweRlaw” library.

We supplemented hypothesis testing with exploratory analysis of regional effects using the Network-Based Statistic Toolbox v1.2 (Zalesky, Fornito, & Bullmore, 2010). This method identifies connected substructures, or components, within the larger network, similar to the cluster-based thresholding approach used in traditional voxel-wise neuroimaging analyses (Zalesky et al., 2010). Permutation testing with 2,000 permutations was then used to determine group differences in components controlling for multiple comparisons using family-wise error (FWE). Because the network-based statistic (NBS) can be less sensitive to focal effects, we also examined regional effects using false discovery rate (FDR; Zalesky et al., 2010). We examined NBS using both extent and intensity; the latter improves the sensitivity of NBS to focal effects (Zalesky et al., 2010).

We also explored the relationship between structure and function. First, network communication measures (e.g., search information of shortest paths, path transitivity) were computed for each pair of nodes in the structural connectivity matrix for each subject. The structural communication measures were then entered into a multiple linear regression model to generate a predicted functional connectivity matrix for each subject (Goñi et al., 2014). In other words, the communication measures for each structural node pair were used as the predictors, and the functional connectivity between that same node pair was used the response. The fitted responses from the linear regression were used to construct the predicted functional connectivity matrix. Finally, a Pearson correlation was computed between the predicted and observed functional matrices for each participant (Goñi et al., 2014). It was unknown how data collected from two different field strengths and/or rodent neurobiology would affect structure-function relationships, so we tested the default communication measures (shortest path length and search information of shortest paths; Goñi et al., 2014) as well as all available measures in the Brain Connectivity Toolbox. These included the default measures plus path transitivity, column-wise z-scored mean first passage time, neighborhood overlap, and matching index (Goñi et al., 2014). These are measures of information flow and community structure that do not require global knowledge of the network’s topology (Goñi et al., 2014; Meghanathan, 2016). Between-group difference in these correlations was measured using two-tailed *t* test.

## RESULTS

### Small-World Organization

**Figure 1.**
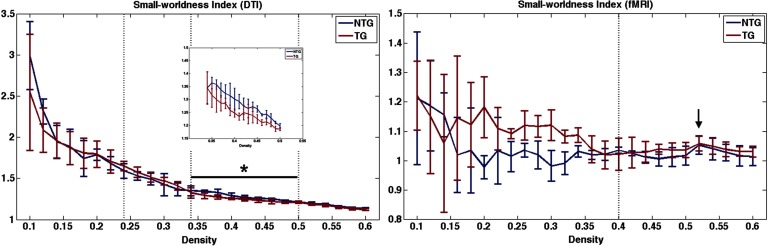
Small-worldness index across network densities. For structural connectomes (DTI), vertical lines indicate area under the curve (AUC) windows. Minimum connection density was 0.24 (first dotted vertical line). At a density of 0.34 (second dotted vertical line), the group curves appear to cross over, and therefore this is where we defined the first AUC window. Maximum density was set at 0.5 (third dotted vertical line) for both modalities based on previous research. For functional connectomes (fMRI), the dotted vertical line indicates minimum connection density (0.4). The black bar and asterisk indicate the significant AUC window, and the inset figure shows the curve on a smaller scale for easier viewing. The arrow indicates the only density where all mice showed small-worldness greater than 1. NTG = nontransgenic; TG = transgenic.

As shown in Figure 1, structural networks demonstrated expected small-world organization defined as a small-worldness index greater than 1 (Humphries & Gurney, 2008) across multiple densities. However, functional networks were small-world for all subjects at only one density (0.52), which was one step above our upper density boundary. Minimum connection density for structural networks occurred at 0.24 and at 0.4 for functional networks.

### AUC Across Densities

**Figure 2.**
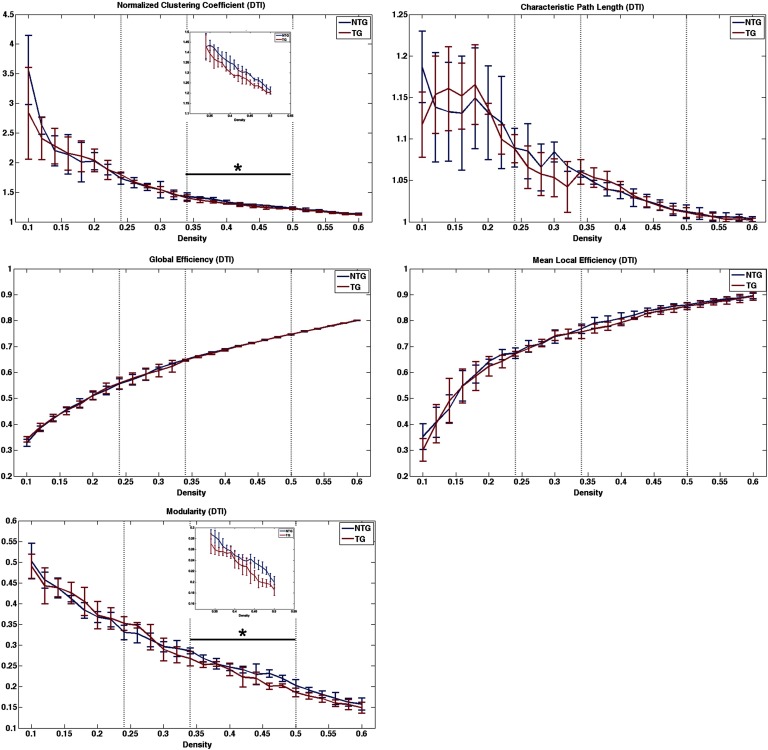
Structural connectome properties. Dotted vertical lines indicate area under the curve (AUC) windows. The black bar and asterisk indicate the significant AUC window, and the inset figure shows the curve on a smaller scale for easier viewing. NTG = nontransgenic; TG = transgenic.

For structural connectomes, permutation testing indicated no significant differences between groups (*p* > 0.19, Figure 2) across the entire range of densities measured (0.24 to 0.5) or across the first density window (*p* > 0.17, Figure 2), which was defined from minimum density to 0.34 where the group curves crossed over. However, transgenic mice demonstrated significantly lower normalized clustering coefficient (*p* = 0.01), small-worldness index (*p* = 0.02), and modularity (*p* = 0.03) compared with nontransgenic mice across the second density window from 0.34 to maximum density (Figures 1 and 2). Module regions are presented in Table 1.

**Table 1.** Module regions

**Nontransgenic**			
**Module 1**	**Module 2**	**Module 3**	**Module 4**
Right amygdala	Left external capsule	Left amygdala	Right brainstem
Right striatum	Right external capsule	Left brainstem	Right central gray
Right cerebellum	Left hypothalamus	Left striatum	Right hypothalamus
Right pallidum	Left superior colliculi	Left central gray	Right midbrain
Right hippocampus	Right superior colliculi	Left cerebellum	
Right inferior colliculi		Left pallidum	
Right neocortex		Left hippocampus	
Left olfactory		Left inferior colliculi	
Right olfactory		Left neocortex	
Right basal forebrain/septum		Left midbrain	
Right thalamus		Left basal forebrain/septum	
		Left thalamus	

**Transgenic**			
**Module 1**	**Module 2**	**Module 3**	
Left external capsule	Right amygdala	Left amygdala	
Right external capsule	Right brainstem	Left brainstem	
Left hypothalamus	Right striatum	Left striatum	
Right hypothalamus	Right central gray	Left central gray	
Left superior colliculi	Right cerebellum	Left cerebellum	
Right superior colliculi	Right pallidum	Left pallidum	
	Right hippocampus	Left hippocampus	
	Right inferior colliculi	Left inferior colliculi	
	Right neocortex	Left neocortex	
	Right olfactory	Left olfactory	
	Right midbrain	Left midbrain	
	Right basal forebrain/septum	Left basal forebrain/septum	
	Right thalamus	Left thalamus	

**Figure 3.**
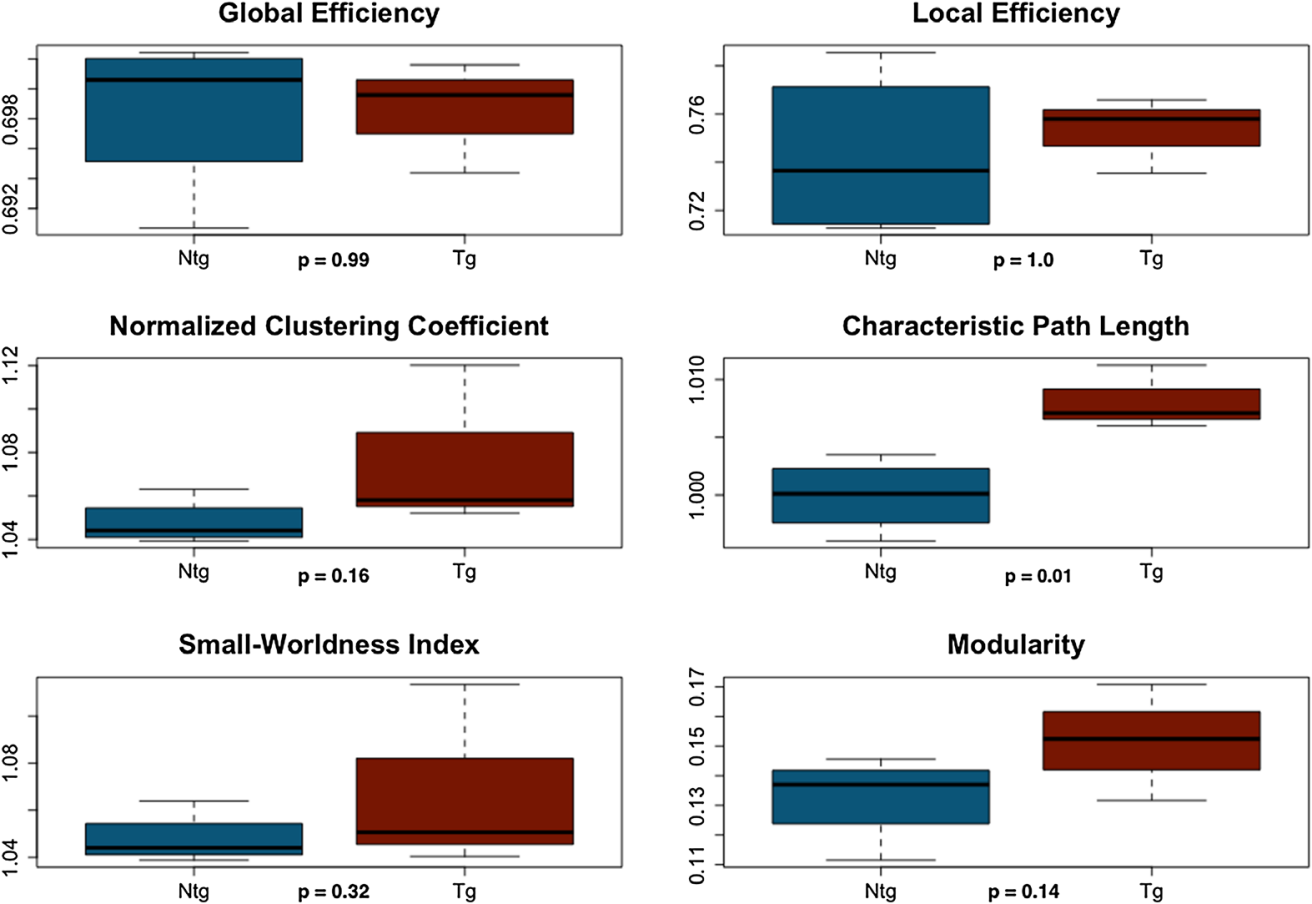
Functional connectome properties at minimum connection density. NTG = nontransgenic; TG = transgenic.

Given the above small-worldness results, we did not compare the AUCs of functional connectomes between groups. It was not possible to simply exclude data since the lack of small-worldness affected different mice at different densities. However, at minimum connection density, connectomes of all subjects but one in the transgenic group showed small-world organization, and therefore we compared connectome metrics at this specific density after excluding the transgenic subject. *T* test indicated significantly higher characteristic path length in transgenic mice (*t* = 3.64 *p* = 0.01, Figure 3).

### Weighted Networks

**Figure 4.**
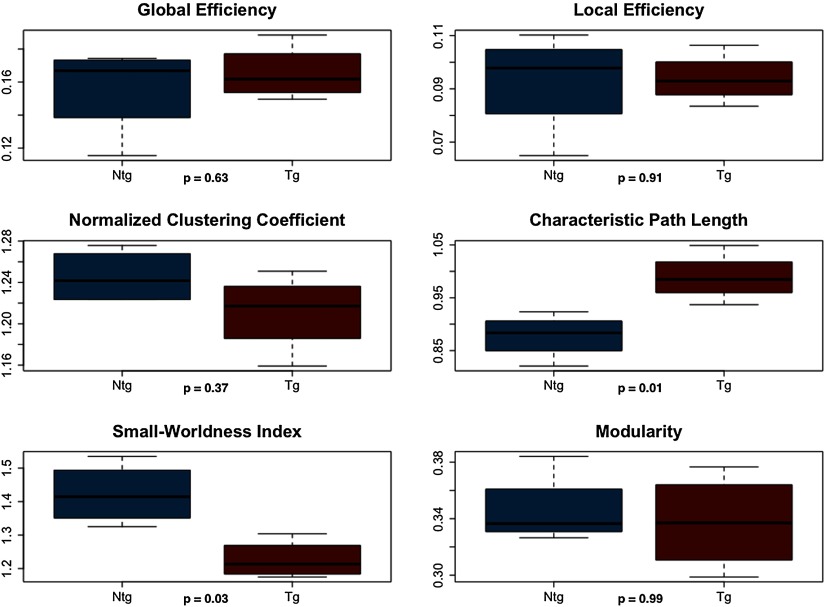
Weighted structural connectome properties. NTG = nontransgenic; TG = transgenic.

All weighted structural networks demonstrated small-world organization. As shown in Figure 4, general linear models covaried for density indicated that structural connectomes of transgenic mice showed significantly higher characteristic path length (*F* = 15.2, *p* = 0.01) and lower small-worldness index (*F* = 9.73, *p* = 0.03) compared with controls.

**Figure 5.**
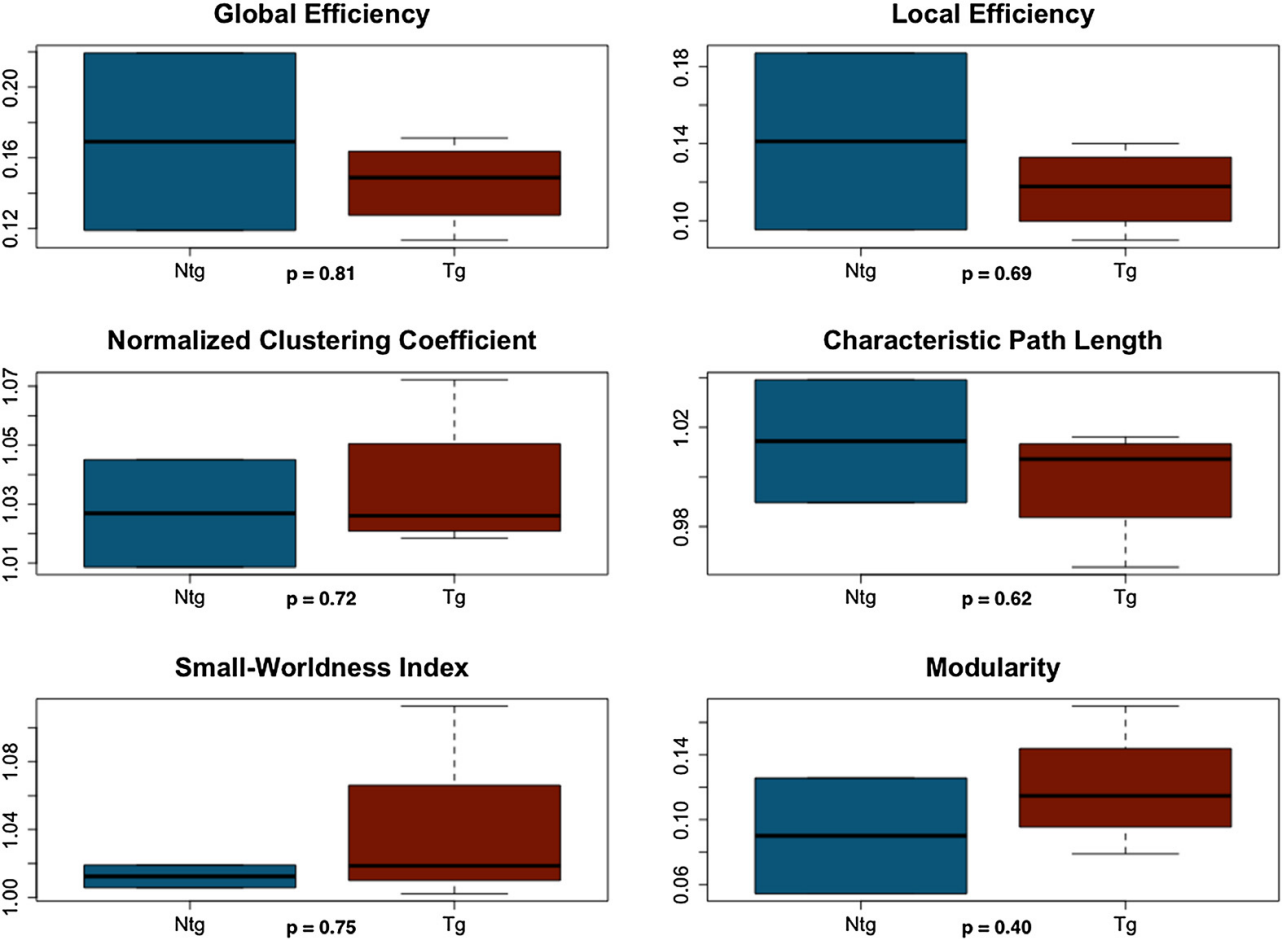
Weighted functional connectome properties. NTG = nontransgenic; TG = transgenic.

Weighted functional connectomes for two nontransgenic mice did not demonstrate small-worldness, so these were excluded. There were no significant group differences (Figure 5).

### Hubs: Degree Distribution

**Figure 6.**
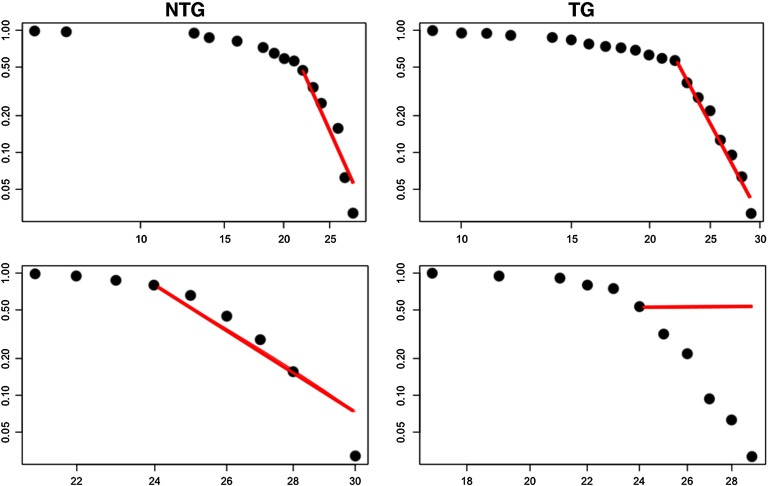
Power-law fit of cumulative degree distributions. Power-law fit is shown as a plot of log degree (x-axis) by log cumulative degree distribution (y-axis). Left column = nontransgenic (NTG), right column = transgenic (TG), top row = weighted structural connectomes, bottom row = weighted functional connectomes.

Both groups showed goodness of fit with the power-law with no between-group difference (*p* = 0.68). There was no significant group difference in power-law fit for either modality at minimum density (*p* > 0.343). For weighted functional networks, the transgenic group showed poor fit with the power-law (Figure 6), and this was significantly lower than that of the control group (*p* < 0.001).

### Regional Connectivity

There were no significant regional effects for structural or functional connectomes weighted or at minimum density via NBS or FDR comparison.

### Relationship Between Structure and Function

**Figure 7.**
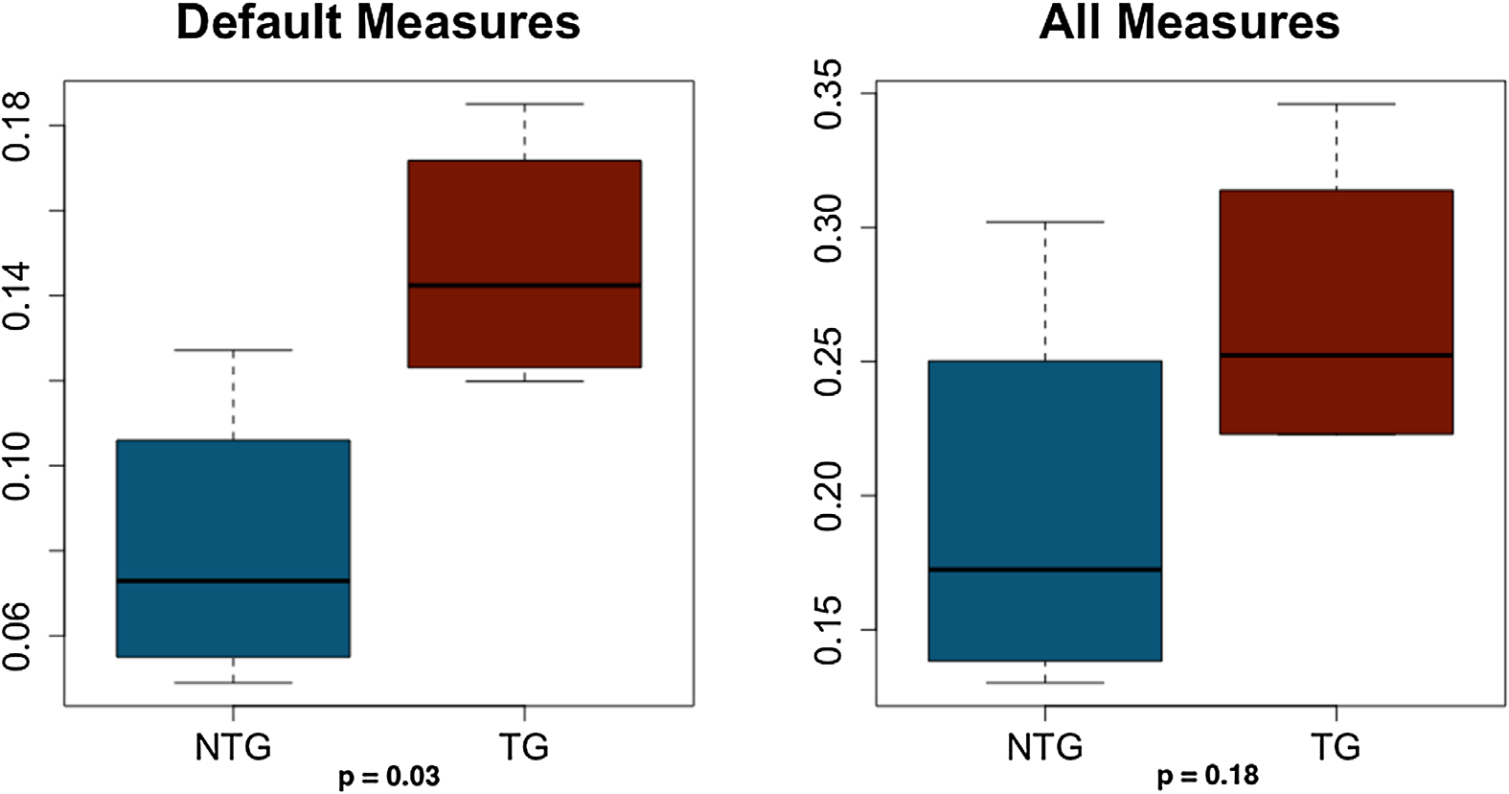
Correlation coefficients for structural and functional connectivity. Default measures = shortest path length, search information of shortest path length; all measures = default measures plus path transitivity, column-wise z-scored mean first passage time, neighborhood overlap, and matching index. NTG = nontransgenic; TG = transgenic.

This analysis was conducted only on weighted networks without excluding any subjects. Using default communication measures resulted in significant correlations between structure and function for five out of eight subjects (*p* < 0.008). Using all communication measures resulted in significant correlations for all subjects (*p* < 0.004). *T* tests indicated that correlation coefficients based on the default model were significantly higher in the transgenic group (*t* = 2.92, *p* = 0.03), but there was no difference in the coefficients from the all-measures model (*t* = 1.53, *p* = 0.18, Figure 7).

## DISCUSSION

To our knowledge, this is the first study to evaluate connectome organization in an AD mouse model. Few if any studies have compared connectomes in rodent models of disease groups. Using in vivo resting-state fMRI and ex vivo DTI, we constructed and measured functional and structural connectomes for 5XFAD transgenic mice. This mouse model is characterized by aggressive amyloid pathology. We evaluated connectome properties in these mice across multiple network densities (i.e., thresholds) and also for weighted, unthresholded networks and compared them with the connectomes of nontransgenic control mice.

Weighted DTI-based structural networks demonstrated significantly higher path length and lower small-worldness index in transgenic mice after controlling for network density. Weighted functional networks also demonstrated higher characteristic path length at minimum connection density. Clinical studies of patients with AD have demonstrated higher characteristic path lengths of DTI- or fMRI-based connectomes (Lo et al., 2010; Wang et al., 2012; Zhao et al., 2012). Increased characteristic path length has also been observed in patients with AD using gray matter structural connectomes (Kim et al., 2016; Pereira et al., 2016) and electroencephalography (EEG)-based networks (Stam et al., 2007). In a small-world network, clustering coefficient is greater than that of random networks, while the path length is comparable to that of random networks (Bassett & Bullmore, 2006; Humphries & Gurney, 2008; Watts & Strogatz, 1998). Characteristic path length is defined as the average shortest path length between all pairs of network nodes divided by the mean path length of benchmark random networks (Bassett & Bullmore, 2006; Humphries & Gurney, 2008; Watts & Strogatz, 1998; Zalesky et al., 2012). Therefore, higher characteristic path length suggests disconnection within the network such that longer, less efficient routes of information exchange must be taken. It also suggests that the network is less random in terms of path length.

We also observed lower normalized clustering coefficient, small-worldness index, and modularity, indicating that the connectomes of transgenic mice were more similar to random networks in these properties. Lower clustering coefficient suggests lower network segregation or specialization, while lower small-worldness index reflects the overall similarity to random networks in that segregation and integration (i.e., path length) are imbalanced (Bassett & Bullmore, 2006; Watts & Strogatz, 1998). Previous DTI and fMRI connectome studies of patients with AD have also demonstrated lower normalized clustering, small-worldness, and modularity (Brier et al., 2014; Sun et al., 2014; Supekar, Menon, Rubin, Musen, & Greicius, 2008).

However, some studies have observed conflicting results, including lower characteristic path length in DTI (Daianu et al., 2013), gray matter (Tijms, Moller et al., 2013), fMRI (Sanz-Arigita et al., 2010), EEG (de Haan et al., 2009), and magneto-encephalogram (MEG; Stam et al., 2009) connectomes in patients with AD. Inconsistencies in connectome findings are a well-known issue in the literature (for reviews, see Dai & He, 2014; Tijms, Wink et al., 2013) and often reflect differences in methodology such as imaging modality and/or choice of thresholding method. This was part of our rationale for a multimodal study including different thresholding methods. Our most consistent findings included higher characteristic path length in transgenic mice, which was noted in weighted, unthresholded DTI connectomes, and fMRI connectomes thresholded to minimum connection density. We also observed lower small-worldness index in transgenic mice in DTI connectomes thresholded to higher densities and in weighted, unthresholded DTI graphs. Small-worldness index is the ratio of clustering coefficient to path length (Bassett & Bullmore, 2006; Watts & Strogatz, 1998) and therefore, lower values can reflect lower normalized clustering and/or higher characteristic path length.

Clinical studies have also noted decreased network efficiency in patients with AD (Daianu et al., 2013; Fischer et al., 2015; Lo et al., 2010; Reijmer et al., 2013), which we did not observe. Efficiency and path length are related measures (Achard & Bullmore, 2007). Since this was a preliminary study, we may have lacked power to detect differences in efficiencies. The 5XFAD mouse model used in this study is associated with accelerated amyloid-beta pathology. Both amyloid-beta and tau are believed to synergistically drive neurodegenerative processes involved in AD (Bloom, 2014; Lloret et al., 2015; Stancu, Vasconcelos, Terwel, & Dewachter, 2014). 5XFAD do not have significant tau pathology. It is possible that greater tau burden and/or some other AD-related neuropathology is more associated with impairments in connectome efficiency. We evaluated the 5XFAD mice at an age prior to significant neuronal and synaptic loss, which may have preserved network efficiency.

Lower cerebrospinal fluid (CSF) levels of amyloid-beta have been associated with higher path length and lower clustering of the gray matter structural connectome in human participants; low levels of amyloid-beta in the CSF indicate higher amyloid plaque burden in the brain (Tijms et al., 2015). Gray matter and DTI-based connectome properties show moderate convergence (Gong, He, Chen, & Evans, 2012). Accordingly, our findings provide indirect support for amyloid-beta effects on connectome properties of the 5XFAD transgenic mouse connectome. We did not have molecular assays available for analysis in this study. Future AD transgenic mouse connectome studies could provide unique insights regarding the molecular mechanisms underlying impairments in various connectome properties associated with AD. For example, Golgi staining and longitudinal fluorescent microscopy could be used to examine neuronal morphology and survival rates in impaired connectome regions. Evaluating the role of mitochondrial dysfunction (Lin & Beal, 2006) via Seahorse flux technology (Brand & Nicholls, 2011) is another potential application.

Previous research has demonstrated lower connectivity among regions involved in specific brain networks of patients with AD, including the default mode network (Bai et al., 2011; Damoiseaux, Prater, Miller, & Greicius, 2012; Lehmann et al., 2013; Simic, Babic, Borovecki, & Hof, 2014). Relevant subnetworks have been shown to be present in mice both structurally and functionally (Liska, Galbusera, Schwarz, & Gozzi, 2015; Stafford et al., 2014), but we did not find any significant regional connectome differences between transgenic mice and controls. This may again reflect limited statistical power and/or may indicate diffuse regional effects. Modularity was lower at higher densities in structural connectomes of transgenic mice, suggesting fewer dissociable networks. Transgenic mice appeared to lack separation between certain sensorimotor/homeostatic regions and other networks.

Both functional and structural connectome topologies showed the expected goodness of fit with a power-law distribution. The power-law fit is believed to reflect the brain network’s hub organization wherein the majority of information processing is handled by a small number of core regions (Achard et al., 2006). This is consistent with other studies showing presence of hubs in the mouse brain (Liska et al., 2015; Rubinov, Ypma, Watson, & Bullmore, 2015). There was no difference in power-law fit between the groups for structural connectomes, but transgenic mice showed a significantly poorer power-law fit in weighted functional connectomes. Previous studies have suggested that AD pathogenesis may selectively target certain hub regions (Dai et al., 2015; Stam et al., 2009; Xie & He, 2011; Yao et al., 2010; Zhou et al., 2012).

Despite problematic functional connectomes that did not show adequate small-world characteristics, functional connectivity was predicted from structural connectivity. Transgenic mice tended to show hyper-correlation of structural and functional networks compared with controls. Such hyper-correlation has been noted in patients with neurologic disorders (Kesler et al., 2017; Rudie et al., 2012; Wirsich et al., 2016). However, Sun et al. (2014) observed lower structure-function coupling in connectomes of patients with AD. Few studies have examined both structural and functional connectomes in AD, and therefore further investigation regarding the relationship between structure and function is required.

There are several limitations to consider for this preliminary, pilot study. It is unclear why functional connectomes failed to demonstrate expected small-worldness. This could be the results of anesthesia, which has been shown to attenuate intrinsic functional networks (Boveroux et al., 2010; Peltier et al., 2005). The effects of anesthesia on connectome organization are currently unknown. MRI field strength could play a role, although a previous study was also conducted at 7 Tesla (Liska et al., 2015). Another drawback is that fMRI and DTI were acquired at different MRI field strengths. This study is also limited by the small sample, which may have reduced our power to detect certain effects. However, we used a conservative statistical approach, including permutation analysis with a large number of permutations and correction for multiple comparisons where appropriate. Currently there is no standard regarding the parcellation scheme for connectome analyses, and therefore a different approach may yield alternate results. DTI-based connectomes have particular limitations, as they have been shown to correspond poorly with neuron tracer data (Calabrese, Badea, Cofer, Qi, & Johnson, 2015). However, as noted above, DTI connectome properties have been shown to differentiate patient groups and therefore seem to provide valuable insights regarding the effects of AD on brain networks.

In conclusion, we demonstrated preliminary evidence that connectome properties of 5XFAD transgenic mice show some correspondence with results observed in patients with AD. There were several innovative aspects of this study, including connectome measurement in an AD mouse model, multimodal connectome measurement, and the use of different network thresholding methods. Future studies in mice could allow us to better understand the molecular mechanisms underlying connectome disruption in AD. These models could also aid in drug discovery and preclinical trials for AD by providing outcome measurements of connectome organization.

## ACKNOWLEDGMENTS

The authors would like to thank the faculty and staff of the MD Anderson Small Animal Imaging Facility as well as Robia Pautler, PhD, and others at the Baylor College of Medicine Small Animal MRI.

## AUTHOR CONTRIBUTIONS

Shelli R. Kesler: Conceptualization; Data curation; Formal analysis; Funding acquisition; Investigation; Methodology; Project administration; Resources; Software; Supervision; Validation; Visualization; Writing – original draft; Writing – review & editing. Paul Acton: Data curation; Methodology; Resources; Writing – review & editing. Vikram Rao: Formal analysis; Methodology; Writing – review & editing. William J. Ray: Conceptualization; Data curation; Funding acquisition; Investigation; Methodology; Project administration; Resources; Supervision; Validation; Visualization; Writing – review & editing.

## FUNDING INFORMATION

This research was funded by the Neurodegeneration Consortium, the MD Anderson Foundation, and the National Institutes of Health (1R03CA191559, 1R01NR014195, 1R01CA172145:SK). The sponsors had no role in the design, implementation, analysis, or interpretation of the study.

## TECHNICAL TERMS

Apolipoprotein E (APOE): A gene that encodes a protein important for the metabolism and transport of fats.
Amyloid precursor protein (APP): A protein believed to be involved in neural development and degeneration.
Presenilin 1 (PSEN1): A gene that encodes a protein involved in processing APP.
Presenilin 2 (PSEN2): A gene that encodes another protein involved in processing APP.
5XFAD transgenic mouse model: A mouse whose DNA has been altered to express five genes that are known to be associated with Alzheimer’s disease.
Connectome: The brain network.
Global efficiency: A measure of network efficiency of information exchange based on path lengths between regions.
Small-world: A network organization or topology associated with high local connectivity and economical large-range connectivity; that is, a balance between segregation and integration.
Modularity: A measure of how well a network can be decomposed into nonoverlapping subnetworks or modules.
