# Supplementary material for: Functional and structural connectome properties in the 5XFAD transgenic mouse model of Alzheimer’s disease
Source: Netw Neurosci. 2018 Jun 1;2(2):241–58. doi: 10.1162/netn_a_00048 (PMC6130552; doi:10.1162/netn_a_00048)

Kesler, S. R., Acton, P., Rao, V., & Ray, W. J. (2018). Supplement to “Functional and structural connectome properties in the 5XFAD transgenic mouse model of Alzheimer's disease.” *Network Neuroscience, 2*(2), 241-258. https://doi.org/10.1162/netn_a_00048

Supplement to Functional and structural connectome properties in the 5XFAD transgenic mouse model of Alzheimer’s disease

Shelli R. Kesler, PhD, Paul Acton, MS, Vikram Rao, MS, William J. Ray, PhD

Supplementary Figure 1. Regions of interest for connectome analysis.

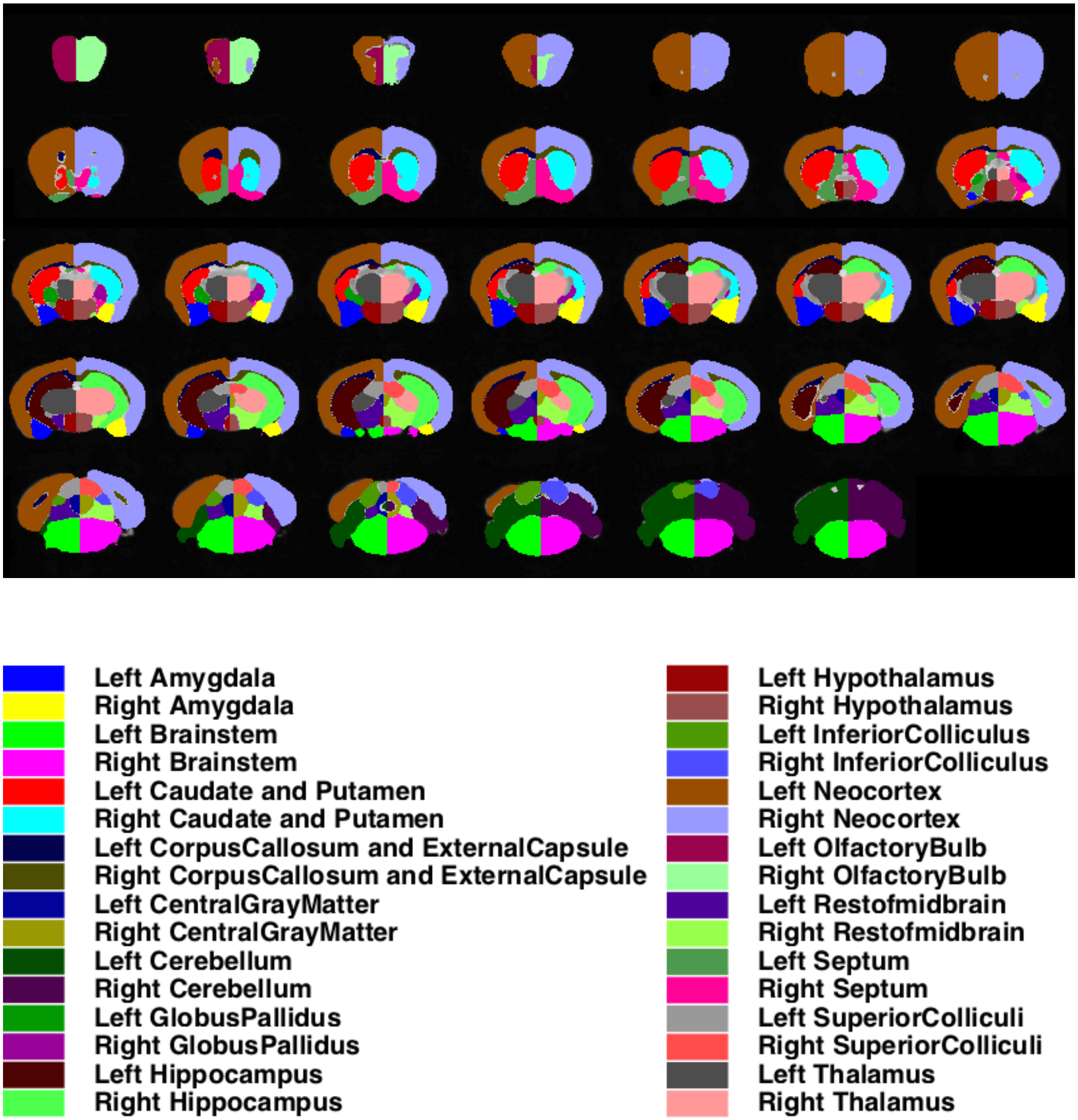

Supplement: Supplementary file 1 [file netn-02-241-s001.pdf]
